# Presence of foodborne pathogens, extended-spectrum β-lactamase -producing *Enterobacteriaceae*, and methicillin-resistant *Staphylococcus aureus* in slaughtered reindeer in northern Finland and Norway

**DOI:** 10.1186/s13028-016-0272-x

**Published:** 2017-01-03

**Authors:** Sauli Laaksonen, Antti Oksanen, Jérôme Julmi, Claudio Zweifel, Maria Fredriksson-Ahomaa, Roger Stephan

**Affiliations:** 1 Department of Veterinary Biosciences, Faculty of Veterinary Medicine, University of Helsinki, Helsinki, Wazama, Finland; 2 Research and Laboratory Department, Production Animal and Wildlife Health Research Unit, Finnish Food Safety Authority Evira, Oulu, Finland; 3 Institute for Food Safety and Hygiene, Vetsuisse Faculty University of Zurich, Zurich, Switzerland; 4 Department of Food Hygiene and Environmental Health, Faculty of Veterinary Medicine, University of Helsinki, Helsinki, Finland

**Keywords:** ESBL-producing *Enterobacteriaceae*, *Listeria monocytogenes*, MRSA, Reindeer fecal samples, *Salmonella*, Shiga toxin genes, *Yersinia*

## Abstract

**Background:**

Various food-producing animals were recognized in recent years as healthy carriers of bacterial pathogens causing human illness. In northern Fennoscandia, the husbandry of semi-domesticated reindeer (*Rangifer tarandus tarandus*) is a traditional livelihood and meat is the main product. This study determined the presence of selected foodborne pathogens, methicillin-resistant *Staphylococcus aureus* (MRSA), and extended-spectrum β-lactamase (ESBL)-producing *Enterobacteriaceae* in healthy semi-domesticated reindeer at slaughter in northern Finland and Norway.

**Results:**

All 470 reindeer fecal samples tested negative for *Salmonella* spp., whereas *L. monocytogenes* was detected in 3%, *Yersinia* spp. in 10%, and Shiga toxins genes (*stx*1 and/or *stx*2) in 33% of the samples. *Listeria monocytogenes* isolates belonged to the serotype 1/2a (14/15) and 4b, *Yersinia* spp. were identified mainly as *Y. kristensenii* (30/46) and *Y. enterocolitica* (8/46), and *stx*2 predominated among the Shiga toxin genes (*stx2* alone or in combination with *stx*1 was found in 25% of the samples). With regard to the frequency and distribution of *stx*1/*stx*2, striking differences were evident among the 10 different areas of origin. Hence, reindeer could constitute a reservoir for Shiga toxin-producing *E. coli* (STEC), but strain isolation and characterization is required for verification purposes and to assess the potential human pathogenicity of strains. On the other hand, the favorable antibiotic resistance profiles (only 5% of 95 *E. coli* isolates were resistant to one or more of the tested antibiotics) and the absence of MRSA and ESBL-producing *Enterobacteriaceae* (when applying selective methods) suggest only a limited risk of transmission to humans.

**Conclusions:**

Healthy semi-domesticated reindeer in northern Finland and Norway can be carriers of certain bacterial foodborne pathogens. Strict compliance with good hygiene practices during any step of slaughter (in particular during dehiding and evisceration) is therefore of central importance to avoid carcass contamination and to prevent foodborne pathogens from entering the food chain.

## Background

In northern Finland, the husbandry of Eurasian tundra reindeer (*Rangifer tarandus tarandus*) is a traditional and economically important livelihood [1]. The total area of reindeer husbandry covers approximately 36% (122,936 km^2^) of Finland (http://paliskunnat.fi/reindeer/reindeer-herding/). The reindeer (about 192,000 animals in the year 2013 [2]) live as semi-domesticated herds, whereby winter corralling and seasonal or permanent supplementary feeding (leading to close animal contacts) is quite common in certain areas. The main product from reindeer husbandry is meat. In the year 2012/2013, approximately 90,000 reindeer were slaughtered in Finland, producing about 2.0 million kilos of meat (http://paliskunnat.fi/reindeer-herders-association/reindeer-info/). Approximately 74% of the reindeer are slaughtered in 19 EU-approved reindeer slaughterhouses. Responsible for the meat inspection and hygiene control are veterinarians working under the lead of the Regional State Administrative Agencies of Lapland. For private consumption and direct marketing, approximately 26% are slaughtered by traditional methods in the field (Regional State Administrative Agencies of Lapland). The European food hygiene legislation (Reg. [EC] No. 852/2004 and Reg. [EC] No. 853/2004) also covers the slaughter and processing of reindeer.

With regard to food-borne diseases, it must be considered that various food-producing animals were recognized in recent years as healthy carriers of important bacterial pathogens causing human illness [3, 4]. Carriage of such zoonotic pathogens in the intestines or on the hides is correlated with the probability of carcass contamination [5]. Hence, if good hygiene practices are not warranted during slaughter, such zoonotic pathogens might enter the food chain by direct or indirect fecal contamination. The aim of this study was to determine the presence of selected bacterial foodborne pathogens, methicillin-resistant *Staphylococcus aureus* (MRSA) and extended-spectrum β-lactamases (ESBL)-producing *Enterobacteriaceae* in healthy semi-domesticated reindeer at slaughter in northern Finland and Norway.

## Methods

### Abattoirs and sample collection

The reindeer in Fennoscandia are free ranging on wide pastures during most parts of the year. In Finland, the migration of reindeer is limited by fences between the cooperatives Supplementary feeding is therefore of growing importance. In the Varang area, the reindeer still have the possibility of natural seasonal migration: during the summer to the coast area and during the winter to the lichen rich mountain area. Under normal weather conditions, supplementary feeding is therefore of minor importance. In autumn round-ups, the reindeer are gathered from pastures and slaughter reindeer are separated from breeding reindeer. Slaughter reindeer are transported to slaughterhouses by vans or trailers and, for longer distances, by special reindeer transport trucks.

During one month (October) of the slaughtering period 2015, 470 healthy (approved in ante mortem inspection) semi-domesticated reindeer calves (aged between 6 and 7 months) were sampled at nine reindeer slaughterhouses in Finland and one in Norway. This age group was selected because the majority of reindeer is slaughtered at about this age and we wanted to assess the potential presence of foodborne pathogens in reindeer at slaughter. Finnish abattoirs were owned by local reindeer herding cooperatives and the butcher staff consisted of trained reindeer owners. The Finnish reindeer were slaughtered in the nearest abattoir and the transport distance by vehicle from the round-up site to the abattoir ranged from 0 to 100 km. The Norwegian abattoir is the major reindeer abattoir in Norway, owned by a private company, and staffed with professional butchers. For the herd of Norwegian reindeer, sampled in this study, the transport distance was about 200 km.

The Finnish abattoirs were medium-sized, EU-approved slaughterhouses with a daily slaughter capacity of 200–400 reindeer. The Norwegian, EU-approved abattoir was bigger with a daily slaughter capacity of at least 700 reindeer. The process and hygiene practices of reindeer slaughter are similar to the slaughter of cattle or sheep. Reindeer are first stunned (bolt pistol), followed by immediate bleeding. Before skinning, the head and distal parts of the legs are removed. Skinning is mainly done using a skinning pulley. Afterwards, reindeer are transferred to the clean part of the abattoir, where evisceration is performed. The cooling of the carcasses starts immediately after slaughtering.

**Fig. 1:**
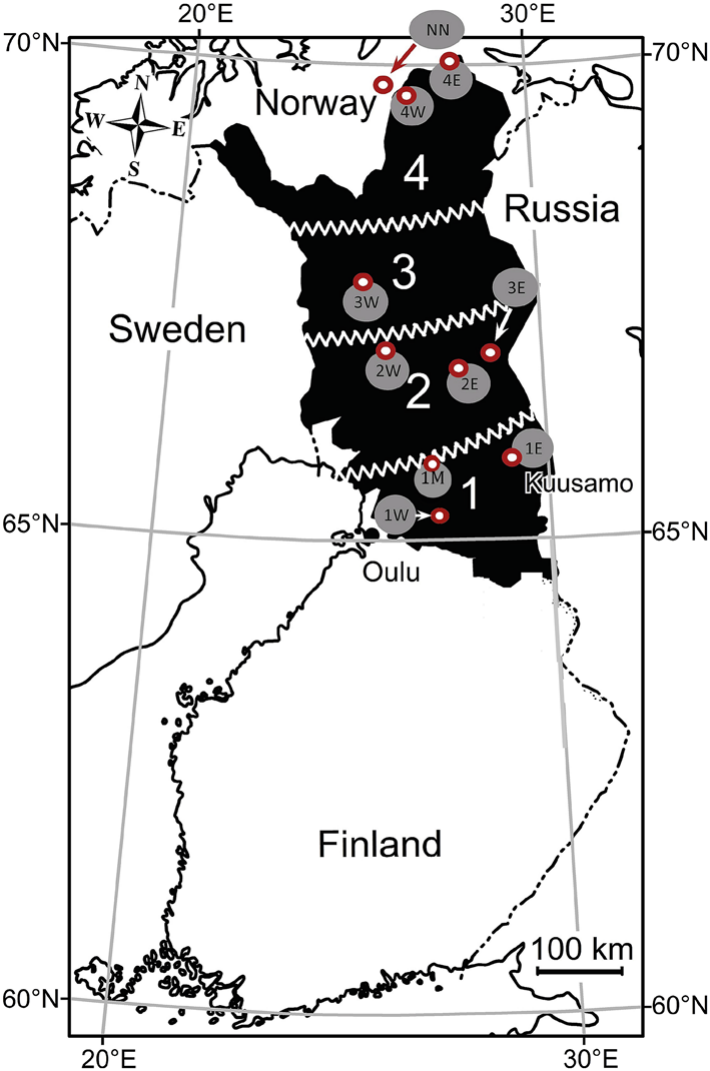
Sampling areas and regions in Finland and in Norway and connected sampling abattoirs. *Black area* Finnish reindeer herding area. Four reindeer herding areas were assigned from south to north (1–4) and cooperatives were named according to their east/west location in the numbered area (*W* West, *M* middle, *E* East). These cooperatives, from south to north were: 1W, 1M, 1E, 2E, 2W; 3E, 3W, 4W, 4E, and northern Norway (NN). *Red circles* are abattoirs and also sampling sites of the areas. The reindeer were transported to the nearest abattoirs

Of the sampled reindeer, 410 originated from northern Finland from nine reindeer herding cooperatives and 60 from the northernmost part of Norway (NN) (Fig. 1; Table 1). The ten geographical areas were thereby equivalent to the ten slaughterhouses mentioned before. The complete reindeer herding area was divided in four areas from south to north (1–4) and cooperatives were named according to their east/west location in the numbered area (W = West, M = middle, E = East). These cooperatives were from south to north: 1W (n = 40); 1M (n = 47); 1E (n = 76); 2E (n = 39); 2W (n = 37); 3E (n = 45); 3W (n = 44); 4W (n = 37); 4E (n = 45); and northern Norway (NN, n = 60) (Fig. 1; Table 1).

**Table 1:** Origin and numbers of sampled reindeer and their transport distances to the abattoirs

Cooperative^a^	No of sampled reindeer	Reindeer density/km2	Transport distance km
*Area 1*			
1W	40	0.76	50
1M	47	1.18	40
1E	76	1.68	20
*Area 2*			
2W	37	1.77	60
2E	39	1.25	40
*Area 3*			
3W	44	1.39	30
3E	45	2.28	100
*Area 4*			
4W	37	2.44	0
4E	45	2.37	0
NN	60	2.70	200

^a^The reindeer herding area was divided in four areas from south to north (1–4) and studied cooperatives were named according to their east/west location in the numbered area (*W* West, *M* middle, *E* East). *NN*, northern Norway

Sampling comprised a total of 34 sampling-days. From each of the 470 examined reindeer, a fecal sample was collected from the large intestine directly after evisceration. Fecal samples were packed into sterile stomacher bags and transported chilled to the Regional Office of Finnish Food Safety Authority (Evira) in Oulu. Samples were frozen and stored at −20 °C up to 2 weeks. In the laboratory, fecal samples were analyzed for *Salmonella* spp., *Listeria monocytogenes*, *Yersinia* spp., and Shiga toxin genes (*stx*). In addition, *Escherichia coli* antibiotic resistance profiles and the occurrence of MRSA and ESBL-producing *Enterobacteriaceae* were assessed.

### *Salmonella* spp

Examination for *Salmonella* spp. was done in accordance with ISO 6579:2007-10 with a modification. A subset of each fecal sample (about 1 g) was enriched (24 h, 37 °C) at a 1:10 ratio in buffered peptone water (BPW; Oxoid, Pratteln, Switzerland). From the first enrichment, 0.1 ml were incubated (24 h, 41.5 °C) in 10 ml of Rappaport–Vassiliadis (RV) broth (Oxoid). The enriched samples (one loopful: approx. 10 µl) were subcultured for 24 h at 37 °C on xylose-lysine-desoxycholate (XLD) agar and mannitol lysine crystal violet brilliant green (MLCB) agar (Oxoid). Presumptive colonies (XLD: red colonies, some with black centers; MLCB: large purple-black colonies, atypical salmonellae grow as mauve-grey colonies and may develop a central black area) were tested for the biochemical properties of *Salmonella* (oxidase reaction, acid production from mannitol, o-nitrophenyl-β-d-galactopyranoside (ONPG) test, H_2_S and indole production as well as urease and lysin decarboxylase activity).

### *Listeria monocytogenes*

Examination for *L. monocytogenes* was done in accordance with ISO 11290-1:2005-01. A subset of each fecal sample (approx. 1 g) was enriched (24 h, 30 °C) at a 1:10 ratio in Fraser broth with half Fraser supplement (Oxoid). From the first enrichment, 0.1 ml were incubated (24 h, 37 °C) in 10 ml of Fraser broth with Fraser supplement (Oxoid). The enriched samples (one loopful: approx. 10 µl) were subcultured for 48 h at 37 °C on chromogenic Ottaviani Agosti agar (bioMérieux, Marcy l’Etoile, France). Presumptive *L. monocytogenes* colonies (turquoise–blue colonies surrounded by an opaque halo on the chromogenic agar) were streaken (24 h, 37 °C) on sheep blood agar (Difco Columbia blood agar base EH, Becton–Dickinson, Allschwil, Switzerland; 5% sheep blood SB055, Oxoid) for evaluation of hemolysis. Isolated *L. monocytogenes* were serotyped using the commercial set of Listeria O-factor and H-factor antisera from Denka Seiken (Pharma Consulting, Burgdorf, Switzerland).

### *Yersinia* spp

A subset of each fecal sample (approx. 1 g) was enriched (24 h, 30 °C) at a 1:10 ratio in BPW (Oxoid). The enriched samples (one loopful: approx. 10 µl) were subcultured for 24 h at 30 °C on cefsulodinirgasan-novobiosin (CIN) agar (Oxoid). Presumptive *Yersinia* colonies (red bull’s-eye surrounded by a transparent border) were streaken (24 h, 37 °C) on sheep blood agar (Difco Columbia blood agar base EH, Becton–Dickinson, Allschwil, Switzerland; 5% sheep blood SB055, Oxoid) and then tested for urease activity. Urease-positive colonies were identified with MALDI-TOF mass spectrometry [6].

### Shiga toxin genes

A subset of each fecal sample (about 1 g) was enriched (18–24 h, 37 °C) at a 1:10 ratio in *Enterobacteriaceae* enrichment (EE) broth (Becton–Dickinson). After incubation (24 h, 37 °C) of the enriched samples on sheep blood agar (Difco Columbia blood agar base EH, Becton–Dickinson; 5% sheep blood SB055, Oxoid), the colonies were washed off with 2 ml of 0.85% saline solution. To screen the samples by real-time polymerase chain reaction (PCR) for *stx*1 and *stx*2, the Assurance GDS^®^ Assay MPX ID for Top STEC (Tq 71019-52; Bio Control Systems, Bellevue, WA, USA) was applied [7]. To compare the frequencies of *stx*1, *stx*2, and both *stx*1 and *stx*2 among fecal samples and the occurrence of Shiga toxin genes among fecal samples of reindeer from different areas of origin, contingency tables (Fisher’s exact test) were used.

### *Escherichia coli* isolation and antibiotic resistance profiles

To assess antibiotic resistance profiles of *E. coli* isolates, about every fifth of the enrichments prepared for the detection of Shiga toxin genes were selected (EE broth; 18–24 h, 37 °C). The enriched samples (one loopful: approx. 10 µl) were subcultured for 24 h at 37 °C on chromogenic RAPID’ *E. coli* 2 agar (Bio-Rad Laboratories, Reinach, Switzerland). One *E. coli* colony (violet to pink on RAPID’ *E. coli* 2 agar) from each of the 95 samples was selected and subjected to susceptibility testing against 12 antimicrobial agents by the disc diffusion method according to the Clinical and Laboratory Standards Institute (CLSI) protocols and criteria [8]. The panel included ampicillin (AM, 10 µg), amoxicillin-clavulanic acid (AMC, 30 µg), chloramphenicol (C, 30 µg), cephalothin (CF, 30 µg), ciprofloxacin (CIP, 5 µg), cefotaxime (CTX, 30 µg), gentamicin (GM, 10 µg), kanamycin (K, 30 µg), nalidixic acid (NA, 30 µg), streptomycin (S, 10 µg), tetracycline (TE, 30 µg), and trimethoprim (TMP, 5 µg) (Becton–Dickinson).

### Methicillin-resistant *Staphylococcus aureus*

Examination for MRSA was done using a two-step enrichment procedure. A subset of each fecal sample (approx. 1 g) was enriched first (18–24 h, 37 °C) at a 1:10 ratio in Mueller–Hinton broth supplemented with 6.5% NaCl (24 h, 37 °C) and subsequently in 5 ml tryptone soy broth (TSB; Oxoid) supplemented with 75 mg/l aztreonam and 5 mg/l cefoxitin (24 h, 37 °C). The enriched samples (one loopful: approx. 10 µl) were then subcultured for 24 h at 37 °C on chromogenic Brilliance MRSA 2 agar (Oxoid). Denim blue colonies on Brilliance MRSA 2 agar are presumptive positive for MRSA.

### ESBL-producing *Enterobacteriaceae*

A subset of each fecal sample (approx. 1 g) was enriched (18–24 h, 37 °C) at a 1:10 ratio in EE broth (Becton–Dickinson). For detection of *Enterobacteriaceae* producing extended-spectrum β-lactamases (ESBLs), the enriched samples (one loopful: approx. 10 µl) were subcultured for 24 h at 37 °C on chromogenic Brilliance ESBL agar (Oxoid).

## Results and discussion

### Presence of foodborne pathogens

To assess the presence of selected bacterial foodborne pathogens, fecal samples from 470 healthy semi-domesticated reindeer were examined for *Salmonella* spp., *L. monocytogenes*, *Yersinia* spp., and Shiga toxin genes at slaughter. Recent data on the presence of bacterial foodborne pathogens in healthy reindeer are limited to very few surveys, mainly originating from Finland and Norway [9–13].

All 470 fecal samples from reindeer tested negative for *Salmonella* spp. (Table 2). However, it must be considered that freezing of fecal samples for storage might have had an effect on bacterial populations, especially on *Salmonella*. *Salmonella* spp. are still a major cause of foodborne diseases and may colonize the intestinal tract of a large number of mammals and birds [3]. But comparable to our results, *Salmonella* were not detected in other studies examining feces from reindeer [9, 10, 12].

**Table 2:** Presence of foodborne pathogens and detection of Shiga toxin genes in fecal samples collected from reindeer at slaughter

No of fecal samples	No. (%) of fecal samples from reindeer testing positive for			
	*Salmonella* spp.^a^	*Listeria monocytogenes* ^b^	*Yersinia* spp.^c^	Shiga toxin genes (*stx*1 and/or *stx*2)^d^
470	0 (0%)	15 (3.2%)	46 (9.8%)	153 (32.6%)

^a^Examination according to ISO 6579:2007-10 mod

^b^Examination in accordance with ISO 11290:2005-01, serotyping (serotype 1/2a, n = 14; serotype 4b, n = 1)

^c^Enrichment in BPW, incubation on CIN agar, species identification with MALDI–TOF MS (*Y. enterocolitica*, n = 8; *Y. intermedia*, n = 1; *Y. kristensenii*, n = 30; *Yersinia* spp., n = 7)

^d^Enrichment in EE broth, screening for *stx*1 and *stx*2 by the Assurance GDS^®^ assay for Shiga toxin genes

*Listeria monocytogenes* were detected in 3.2% of the 470 reindeer fecal samples (Table 2). The positive animals originated from six of ten different areas (1W, n = 1; 1M, n = 1; 1E, n = 1; 2E, n = 1; 3W, n = 5; 4W, n = 6). In contrast, Aschfalk et al. [9] did not isolate *Listeria* spp. from Norwegian reindeer, but only 35 fecal samples from cadavers were examined and the isolation procedure was different. *Listeria monocytogenes* as a foodborne pathogen has the potential to cause serious and life-threatening conditions (including septicemia, meningitis, meningoencephalitis, and abortion) in persons with reduced immunity [3, 14]. Of the 15 *L. monocytogenes* isolates from reindeer, 14 belonged to the serotype 1/2a and one to the serotype 4b. Serotype 1/2a strains are frequently found in ready-to-eat food and food-processing environments [15, 16]. Human clinical cases are frequently associated with strains of serotypes 1/2a, 1/2b, and 4b and infections due to serotype 1/2a strains have increased in recent years [15, 17].

*Yersinia* spp. were detected in 9.8% of the 470 fecal samples from reindeer (Table 2). The positive animals originated from nine of ten different areas (1W, n = 1; 1M, n = 14; 1E, n = 20; 2E, n = 3; 2W, n = 1; 3E, n = 1; 3W, n = 4; 4E, n = 1; NN, n = 1). The species of 39 *Yersinia* isolates was identified by MALDI-TOF mass spectrometry: *Yersinia kristensenii* (n = 30), *Y. enterocolitica* (n = 8), and *Y. intermedia* (n = 1). Similarly, in the survey of Kemper et al. [10], 108 (4.8%) *Yersinia* isolates were obtained from reindeer and *Y. kristensenii* (n = 72) and *Y. enterocolitica* (n = 29) predominated. *Yersinia kristensenii* have been isolated from environmental samples, animals, foods, and humans, but their impact on human health remains controversial [18, 19]. On the other hand, although not all *Y. enterocolitica* are considered zoonotic agents and pathogenic for humans, certain strains of *Y. enterocolitica* (some biotypes/serotypes) may cause acute gastroenteritis, reactive arthritis, or mesenteric lymphadenitis and terminal ileitis mimicking appendicitis in humans [3, 20].

Shiga toxin-producing *E. coli* are responsible for a number of human (foodborne) illnesses including diarrhea, hemorrhagic colitis, and the life-threatening hemolytic uremic syndrome (HUS) [3, 21, 22]. The production of one or more Shiga toxins (Stx1, Stx2, and variants) characterizes STEC. However, it must be emphasized that the detection of Shiga toxin genes in fecal samples (without strain isolation and characterization), as performed in the present study, can only be regarded as presumptive presence of STEC. In the examined reindeer fecal samples, genes for Shiga toxins (*stx*1 and/or *stx*2) were detected in a remarkable prevalence of 32.6% (Table 2) and *stx*2 predominated (P < 0.05). Alone or in combination with *stx*1, *stx*2 was found in 24.5% of the samples (Table 3). With regard to 10 different areas of origin, striking differences were evident for the frequency and distribution of *stx*1/*stx*2 (Table 3). The overall highest frequency was found among samples from 2E (P < 0.05; 100%, mainly *stx*1), followed by 4W (54%, only *stx*2) and 2W (46%, only *stx*2), while the lowest frequencies were detected in samples from 1W (P < 0.05; 5%, only *stx*2), NN (12%, only *stx*2), and 3E (13%, only *stx*2). Moreover, compared to the other areas, the predominance of *stx*1 in samples from 2E (P < 0.05) and of both *stx*1 and *stx*2 in samples from 1E (P < 0.05) was striking.

**Table 3:** Detection of Shiga toxin genes (*stx*1/*stx*2) in fecal samples from reindeer from different areas of origin in northern Finland and Norway

Area of origin	No. of fecal samples	No. (%) of samples testing positive for			
		*stx*1 (only)	*stx*2 (only)	*stx*1 and *stx*2	*stx* total
1W	40	0 (0%)	2 (5.0%)	0 (0%)	2 (5.0%)
1M	47	1 (2.1%)	13 (27.7%)	2 (4.3%)	16 (34.0%)
1E	76	0 (0%)	7 (9.2%)	18 (23.7%)	25 (32.9%)
*1 Total*	*163*	*1 (0.6%)*	*22 (13.5%)*	*20 (12.3%)*	*43 (26.4%)*
2E	39	37 (94.9%)	0 (0%)	2 (5.1%)	39 (100%)
2W	37	0 (0%)	17 (45.9%)	0 (0%)	17 (45.9%)
*2 Total*	*76*	*37 (48.7%)*	*17 (22.4%)*	*2 (2.6%)*	*56 (73.7%)*
3E	45	0 (0%)	6 (13.3%)	0 (0%)	6 (13.3%)
3W	44	0 (0%)	8 (18.2%)	1 (2.3%)	9 (20.5%)
*3 Total*	*89*	*0 (0%)*	*14 (15.7%)*	*1 (1.1%)*	*15 (16.9%)*
4W	37	0 (0%)	20 (54.1%)	0 (0%)	20 (54.1%)
4E	45	0 (0%)	12 (26.7%)	0 (0%)	12 (26.7%)
*4 Total*	*82*	*0 (0%)*	*32 (39.0%)*	*0 (0%)*	*32 (39.0%)*
Finland total	410	38 (9.3%)	85 (20.7%)	23 (5.6%)	146 (35.6%)
Northern Norway	60	0 (0%)	7 (11.7%)	0 (0%)	7 (11.7%)
*Total*	*470*	*38 (8.1%)*	*92 (19.6%)*	*23 (4.9%)*	*153 (32.6%)*

Reindeer might become colonized with STEC directly from contact with carrier animals or indirectly from contaminated feed or soil. The colonization pressure could thereby be influenced by the husbandry conditions (e.g., animal density of reindeer, supplementary feeding in corrals, duration of transport and of round-up events before slaughter). A high or low animal density of reindeer might have an impact on the colonization pressure (amount of fecal contamination of the pastures). In the present study, area 1W (Fig. 1) showed the lowest animal reindeer density and the lowest *stx* prevalence.

For most of the sampled reindeer herds, animals are supplementary fed in corrals or on pastures during the winter months. If feeding hygiene is poor and accumulated feces can contaminate the provided fodder, this may increase the colonization pressure. In the areas 1–3 (Fig. 1) reindeer are intensively supplementary fed, mostly in corrals. This may partly explain the high prevalence in some cooperatives. An exemption is the area 3E, where reindeer do not receive any supplementary feeding.

In the area 3E and also in northern Norway (NN) (Fig. 1), the strategy of seasonal translocation between summer and winter pasture is applied. Hence, reindeer are periodically moved to other pastures, on which they have not grazed before. On these pastures, there is less (fresh) fecal contamination pressure. This could partly explain the low *stx* prevalence in the area 3E and NN areas.

Transport distances of reindeer to the abattoirs varied in the present study from zero (reindeer walk from round-up corral to the abattoir; areas 4W and 4E) to about 200 km (NN) (Table 1). Depending on transport distances and durations, reindeer are usually allowed to rest (1–12 h) in fences at the abattoirs before slaughter. Drinking water is freely available and reindeer must be fed if the waiting time exceeds 12 h (Finnish Animal Protection Regulation 7.6.1996/396). In the present study, we did not find any connection between transport distances/durations and the observed *stx* prevalence.

Furthermore, the colonization pressure could be increased by the corralling of large numbers of reindeer before slaughter as well as stressful handling and long durations of round-ups before slaughter (occasionally up to three days). Such aspects could be of particular importance in the areas 4W and 4E.

Although detection of Shiga toxin genes in fecal samples only indicates the presumptive presence of STEC, our results suggest that healthy semi-domesticated reindeer in northern Finland and Norway, as other wild ruminants [23], could probably constitute a reservoir for STEC. However, further investigations (strain isolation and characterization) are required for verification purposes. If reindeer are confirmed as a STEC reservoir, the potential human health risk associated with STEC from reindeer must be evaluated [24]. Further, the risk of STEC transmission from reindeer to humans, by consumption of undercooked meat or other food contaminated by feces, as well as to livestock when sharing pastures with reindeer, must not be neglected. In contrast to our findings, STEC were not or only rarely detected in reindeer in previous studies [9–13] and the few positive *E. coli* isolates harbored *stx*1 [9, 10]. However, direct comparison of our results with the literature is strongly hampered by varying detection procedures applied, and that two surveys [11, 13] focused mainly on *E. coli* O157.

### Antibiotic resistance

Disc diffusion tests showed low levels of antibiotic resistances: only five (5.3%) isolates were resistant to one or more of the tested antibiotics. However, it must be considered that clinical breakpoints were used (which could show a lower proportion of resistance than the application of epidemiological cut-offs). One strain showed resistance to ampicillin, amoxicillin with clavulanic acid, cephalothin and cefotaxime, two strains were resistant to streptomycin and tetracycline, while the two remaining strains were resistant to ampicillin or cephalothin. Thereby, the single *E. coli* isolate resistant to cefotaxime could be an ESBL-producer, but this needs further confirmation. Interestingly, Lillehaug et al. [12] reported more resistant *E. coli* in wild reindeer (24%) than in other hunted wild cervids (2.2%).

With regard to antibiotic resistance, methicillin-resistant staphylococci, in particular MRSA, and ESBL-producing *Enterobacteriaceae* are currently of special concern. In recent years, it has been widely recognized that the dissemination of MRSA and ESBL-producing bacteria is an issue no longer restricted to the medical/health care system [25–28]. In our study, applying selective methods, no MRSA or ESBL-producing *Enterobacteriaceae* were detected among the 470 fecal samples from healthy reindeer. Hence, in contrast to livestock, a favorable situation with regards to antimicrobial resistance is present in the reindeer population.

## Conclusions

Healthy semi-domesticated reindeer in northern Finland and Norway can be carriers of bacterial foodborne pathogens. In particular, reindeer could constitute a reservoir for STEC, but strain isolation and characterization is required for verification and to assess the potential human pathogenicity. Strict compliance with good hygiene practices during any step of slaughter (in particular during skinning and evisceration) is therefore of central importance to avoid carcass contamination and to prevent foodborne pathogens from entering the food chain.
